# Impact of Precision in Staging Acute Kidney Injury and Chronic Kidney Disease on Treatment Outcomes: An Observational Study

**DOI:** 10.3390/diagnostics14222476

**Published:** 2024-11-06

**Authors:** Olga Endrich, Christos T. Nakas, Karen Triep, Georg M. Fiedler, Jaime J. Caro, Alistair McGuire

**Affiliations:** 1 Medical Directorate, Inselspital, Bern University Hospital, 3010 Bern, Switzerland; karen.triep@insel.ch; 2 University Institute of Clinical Chemistry, University of Bern, Inselspital, Bern University Hospital, 3010 Bern, Switzerland; cnakas@uth.gr (C.T.N.); martin.fiedler@insel.ch (G.M.F.); 3 Department of Health Policy, London School of Economics, London WC2A 2AE, UK; j.caro@lse.ac.uka.j.mcguire@lse.ac.uk (A.M.); 4 Laboratory of Biometry, University of Thessaly, 38446 Volos, Greece; 5 Department of Epidemiology, Biostatistics and Occupational Health, McGill University, Montreal, QC H3A 1G1, Canada

**Keywords:** data-driven diagnosis, electronic health records, digital markers, rule engine, precision medicine, real-world data, kidney disease, KDIGO staging

## Abstract

(1) Background: “Kidney Disease: Improving Global Outcomes” (KDIGO) provides guidelines for identifying the stages of acute kidney injury (AKI) and chronic kidney disease (CKD). A data-driven rule-based engine was developed to determine KDIGO staging compared to KD-related keywords in discharge letters. (2) Methods: To assess potential differences in outcomes, we compare the patient subgroups with exact KDIGO staging to imprecise or missing staging for all-cause mortality, in-hospital mortality, selection bias and costs by applying Kaplan–Meier analysis and the Cox proportional hazards regression model. We analysed 63,105 in-patient cases from 2016 to 2023 at a tertiary hospital with AKI, CKD and acute-on-chronic KD. (3) Results: Imprecise and missing CKD staging were associated with an 85% higher risk of all-cause and in-hospital mortality (CI: 1.7 to 2.0 and 1.66 to 2.03, respectively) compared to exact staging for any given disease status; imprecise or missing AKI staging increased in-hospital mortality risk by 56% and 57% (CI: 1.43 to 1.70 and 1.37 to 1.81, respectively) in patients with AKI. (4) Conclusions: Exact staging is associated with better outcomes in KD management. Our study provides valuable insight into potential quality and outcome improvements and lower costs, considering elderly patients, women and patients with acute-on-chronic KD as the most vulnerable.

## 1. Introduction

Global priorities are changing to an increased focus on non-communicable diseases (NCD). Kidney disease (KD) is one of the ten most important fastest-growing drivers of burden [1] and a significant contributor to mortality and disability. However, the uncertainty of obtaining a baseline and KD’s mostly asymptomatic progression complicates the diagnostic and staging process. In 2017, 697.5 million cases of all-stage CKD were recorded worldwide, with a global prevalence of 9.1% (8.5 to 9.8) [1,2]. Neuen describes the importance of CKD for the global NCD agenda [3]. According to the Burden of Disease scenarios for 204 countries and territories, a forecasting analysis for the Global Burden of Disease Study 2021, diabetes and kidney disease are the two most extensive Level 2 causes of the increase in the number of disability-adjusted life years (DALYs), from 117.3 million (103.7–134.3) in 2022 to 240.1 million in 2050 (192.6–287.8) [4].

Historically, more than 35 definitions of KD have been proposed, reflecting the difficulty of capturing the disease pattern; the uncertainty of obtaining a baseline and the mostly asymptomatic progression complicates the staging process [5]. Considering the underlying physiological processes of acute kidney injury (AKI) and chronic kidney disease (CKD) as a unified and bidirectional clinical syndrome facilitates effective prevention and treatment strategies [6,7,8,9,10]. In 2012, the “Kidney Disease: Improving Global Outcomes” (KDIGO) clinical practice guideline harmonised these definitions into one universal set of criteria [5].

The definition of KD requires a multi-level analysis of a patient’s recent and historical laboratory values. If conducted manually, the calculation is prone to errors and time-consuming, often aggravated by the baseline uncertainty. Although it is possible to detect KD using routine laboratory data, many clinicians currently fail to assess KD correctly.

Why might it be essential to assess the KD stage correctly? The treatment approach for kidney disease varies significantly depending on the stage; correctly assessing the staging helps monitor and prevent complications and disease progression. KDIGO provides recommendations for KD management based on the stage. For AKI, actions such as discontinuing nephrotoxic agents, avoiding radiocontrast procedures, ensuring sufficient volume status and perfusion pressure, monitoring kidney function and timely consideration of Intensive Care Unit (ICU) admission and Renal Replacement Therapy (RRT) are connected to the defined stages of functional impairment. These actions can be supported by using Clinical Decision Support Systems (CDSSs). Multiple clinical practice guidelines for early identification, treatment and the beneficial usage of CDSSs are available [11,12,13,14].

Decisions made by health professionals influence disease progression and survival [7,15,16,17,18,19]. Considering the high costs of treatment of KD and KD-associated complications, the incentive to reduce the risk of complications and progression of the disease is high. Internationally, early detection, including proteinuria or microalbuminuria screening, is considered cost-effective [11,20,21,22,23,24,25,26,27]. In the UK, a mandatory CDSS within all acute National Health Service (NHS) trusts was introduced in 2015 to improve the detection, alerting and response to AKI. This intervention led to a 22% improvement in hospital AKI 3 recoveries [28]. The NHS has published numerous guidelines for detecting and treating KD and has made best practice recommendations [12].

CDSSs often need to be more comprehensive. The usefulness of the existing tools and health care standardisation might be perceived as controversial or constraining or considered a countertrend to personalised medicine. On the other hand, the current decision-making process needs to be better understood, and the consequences of the ad hoc approach need to be uncovered.

Although standardised staging is widely accepted in different fields of medicine, the currently observed KD staging process is more intuition-driven and, to some extent, random. In 2018, our hospital developed and validated a near-real-time data-driven rule engine (“DDD” for Data-Driven Diagnosis) to support the clinical diagnostic and staging processes. The main goal of the DDD rule engine was to implement the compulsory, automatised KDIGO classification of KD based on routinely administrated laboratory measurements [29]. Glomerular filtration rates (eGFRs CKD-EPI 2009 equation [30]), plasma/serum creatinine (SCr) and the albumin-to-creatinine ratio (ACR) were used to calculate an individual baseline and incremental change for each patient treated.

According to the KDIGO Clinical Practice Guidelines (2012) [31], the AKI stages are defined as follows:

- Stage 3: increase in SCr from under 4 mg/dL (353.6 µmol/L) to over 4 mg/dL within 7 days;
- Stage 3: increase in SCr by 200% or more within 7 days;
- Stage 2: increase in SCr by 100–200% within 7 days;
- Stage 1: increase in SCr by 50–100% within 7 days;
- Stage 1: increase in SCr by 0.3 mg/dL (26.52 µmol/L) within 48 h.

CKD is defined according to the KDIGO Clinical Practice Guideline for the Evaluation and Management of CKD (2012), i.e., a decreased GFR of <60 mL/min/1.73 m^2^, an albumin creatinine ratio (ACR) of >30 mg/g (>3 mg/mmol) or a history of kidney transplantation (estimated according to the CKD-EPI equation). GFR categories are assigned as follows:

- Stage 5: all values under 15 mL/min/1.73 m^2^ for >91 days;
- Stage 4: all values under 30 mL/min/1.73 m^2^ for >91 days;
- Stage 3: all values under 60 mL/min/1.73 m^2^ for >91 days;
- Stage 2: all values under 90 mL/min/1.73 m^2^ for >91 days.

The KDIGO guidelines do not specifically define a baseline; however, several methods were compared for baseline estimation [31,32]. In our approach, we defined the baseline value as either the lowest value during hospitalisation or the arithmetic mean of all available SCr measurements 90 days before the index admission.

Albuminuria is integrated into the KDIGO staging as a marker of kidney damage and has an essential prognostic value; the ACR values are defined as follows:

- Severely increased: SCr (µmol/L)/albumin (g/L) > 30 mg/g;
- Moderately increased: SCr (µmol/L)/albumin (g/L) between 3 and 30 mg/g;
- Normal to mildly increased: SCr (µmol/L)/albumin (g/L) < 30 mg/g.

The rule engine was designed to meet the criteria for certification to the software standards for medical devices. The validation included a testing with synthetic data (Phase 1), with an annotated real-world data set (Phase 2) and a proof-of-concept testing (Phase 3). The outputs of the rule engine were presented in a confusion matrix and used for release approval, aiming for 100% accuracy in identifying true positive and true negative cases.

The initial goal of a near-real-time alert system has yet to be realised, primarily due to the generally slow adoption of CDSSs in Switzerland, which means that the DDD rule engine is disconnected from the clinical treatment process. As of 2018, this rule engine has been used productively to identify, validate and encode the diagnoses retrospectively after a patient’s discharge using the International Classification of Diseases, German Modification (ICD-10 GM). A list of the ICD-10 GM diagnoses and the corresponding KDIGO KD stages is available in Supplementary File S1.

**Figure 1:**
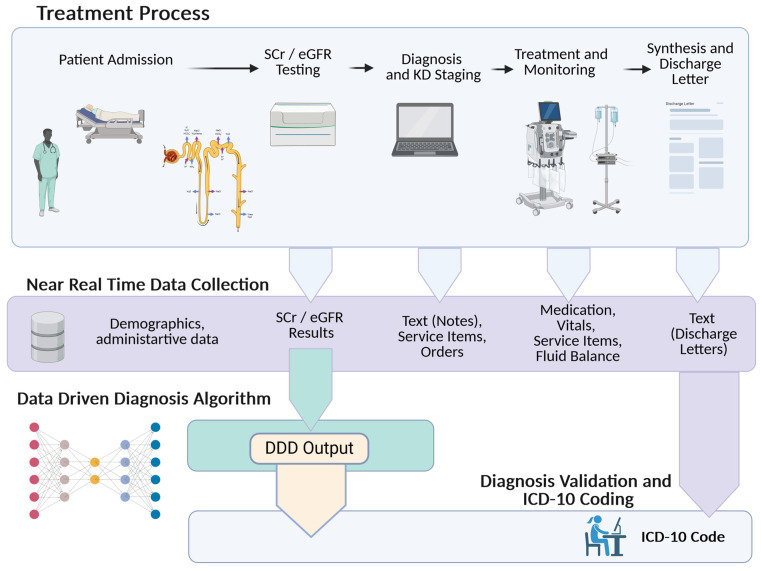
The current process of assessment, treatment, documentation and coding of KD in cases of in-patient hospitalisation. Created in BioRender. Endrich, O. (2024) [33].

The coding experts review and validate each diagnosis generated by the rule engine by cross-referencing the information from the electronic health record system (EHR). If necessary, they also confirm the diagnosis with the clinician. A documented treatment, such as medication, volume management or RRT, is required for a diagnosis to be encoded. ICD coding is performed by certified coding experts and governed by guidelines revised annually by the Swiss Federal Office of Statistics. Encoding is based on discharge letters, all available hospital documents and information and the output of the DDD rule engine. According to the insurance law, the national governmental tariff body requires an annual audit of coded data to prevent upcoding and ensure coding consistency. The entire process, from patient admission to the encoded diagnosis, is visualised in Figure 1. During the last seven years, the DDD rule engine substantially enhanced the quality of encoded ICD-10 diagnoses for KD and created a valuable baseline for the true prevalence of KD in our hospital [29]. In 2018, an observational study was initiated and approved to analyse the impact of DDD-supported staging (ethic approval BASEC-Req-2018-01184).

The main research objective of our retrospective observational study was to understand how KDIGO staging may impact the treatment and outcome. If the decision-making process can be influenced when considering a potential kidney disease, ordering a KD-specific laboratory test and documenting an explicit KD stage might be a digital marker of such consideration. Precise staging may lead to earlier detection, guideline-aligned treatment and rigorous monitoring of laboratory values and may also prevent iatrogenic events such as nephrotoxic medication and contrast-agent-induced kidney function damage. In general, we cannot observe the intention or the decision-making process, but the realisation of the treatment choices is observable and can be analysed using digital markers.

To assess potential differences in outcomes, we compare the patient subgroups with exact KDIGO staging to imprecise or missing staging for all-cause mortality, in-hospital mortality, selection bias and costs. If precise staging is associated with a better outcome, it presents a strong case for implementing a CDSS and using KDIGO staging as a clinical tool to promote patient safety and better outcomes.

## 2. Materials and Methods

Our observational study was started in 2018 to detect the potential impacts of the rule engine on the patients’ individual trajectories and the hospital’s population. The analysis used routine data collected from patients who did not refuse general consent (90% of all patients treated).

The clinical and administrative data for all patients treated are available in the Insel Group Clinical Data Warehouse CDWH. The DDD Kidney Disease rule engine output is stored in a defined data mart and updated daily. The patient’s civil status (alive, deceased) is automatically updated weekly by the Swiss Unique Person Identification Registry (UPI). The hospital costs accounting is carried out using the standardised method RECOLE. The outputs of the existing rule engine, episodes of hospital care and relevant attributes and data points are validated and prepared for extraction.

Data Extraction Clinical Data Warehouse: Inclusion criteria: predefined routinely collected data and DDD output were extracted on 27 March 2024 from all inpatient cases discharged from 1 January 2016 to 31 December 2023 with a coded diagnosis of KD (ICD-10 N17, N18, N19), 18 years or older by hospitalisation, who did not refuse general consent.

KD staging: AKI staging corresponds to ICD-10 codes N17.x, CKD staging to the ICD-10 codes N18.x and KD not specified to N19; details can be found in Supplementary File S1.

Digital marker: A text analysis approach was used to identify the KD staging in the clinical documentation, generally in discharge letters, stored in the CDWH. The KD staging digital marker was defined as *exact CKD*: ‘G’ + g + ‘A’ + str(a), for g in [‘1’,‘2’,‘3’,‘3a’,‘3b’,‘4’,‘5’]; as “*exact* AKI KDIGO”: ‘AKI’ + n + space + g’, for g in [‘1’,‘2’,‘3’,‘I’,‘II’,‘III’,‘l’,‘ll’,‘lll’], for n in ['','N'], for space in ['',' '], while following the KDIGO definition. The KD staging *imprecise* contains different kidney-related terms (Supplementary File S2). The *no* staging was applied if none of the KD staging imprecise terms were found. A fuzzy search algorithm was applied for terms with a tilde character (“~”) to ensure that terms with more than one spelling were found. The extraction used TheFuzz and RapidFuzz libraries and the packages numpy and panda, release 23.3.1, Python, Visual Studio September 2023, version 1.83.

The contrast media application was derived from the CDWH, defined as the combination of the WHOCC ATC code V08 “contrast media” and case-specific individually documented diagnostic or therapeutic imaging service items [34]. The hospitalisation-related application of the nephrotoxic substances per case was derived from the CDWH, including antibiotics, NSAR, chemotherapeutics and other agents by the WHO ATC code [15]. The treatment groups were applied according to the Swiss national method for the hospital planning groups [35]. The Elixhauser van Walraven unweighted and weighted indices were derived based on the selection of the coded ICD-10 diagnoses [36].

Generalised linear modelling methods and the Cox model were used for inference and for the assessment of the effects of covariates of interest and possible confounders on survival according to KD status and KDIGO staging. P-values less than 0.05 were considered statistically significant.

The analysis and reporting follow the STROBE and economic modelling recommendations [37,38]. The study-specific data are stored in a separate SQL data mart embedded in the Clinical Data Warehouse, Insel Group. Data analysis was performed using R, version 4.3.2 (The R Foundation for Statistical Computing, Vienna, Austria), and Stata 16.0 (StataCorp LLC, College Station, TX, USA).

## 3. Results

### 3.1. Descriptive Statistics of the Hospital’s Cohort

Three groups were built to classify the patients’ status of KD: acute kidney injury without a known history of chronic kidney disease (AKI_noCKD), chronic kidney disease (noAKI_CKD) without superimposed episodes of acute kidney injury and acute-on-chronic kidney disease (AKI_CKD). The distribution of the patient’s status of KD, including the KDIGO stages for AKI and CKD and KD staging status (exact AKI, exact CKD, imprecise and no staging) in the hospital’s cohort, are summarised in Table 1.

Most cases (61.5%) were classified as CKD without AKI episodes. The patients in the AKI group were 8–9 years younger, received more potentially nephrotoxic medication and contrast imaging, and were more often admitted to the ICU compared to patients with CKD. In all the KD groups, men were more frequently affected than women; 60% were affected on average. The patients in the acute-on-chronic groups had the highest comorbidity score. Out of all patients, 29% were staged with an *exact AKI* stage and 7% with *exact CKD*; *no* staging was identified in 12% (AKI) and 11% (CKD) of cases, indicating that any keywords related to KD diagnosis were missing in the discharge letter. The KDIGO staging was *imprecise* for most cases (59% for AKI and 81% for CKD). Table 2 shows the distribution of the most relevant treatment groups. Most cases (38%) were allocated to the general medicine treatment group, followed by cardiology (13%) and the musculoskeletal system (6%), showing consistent patterns across KD groups.

### 3.2. Survival Analysis of KD Stages

**Figure 2:**
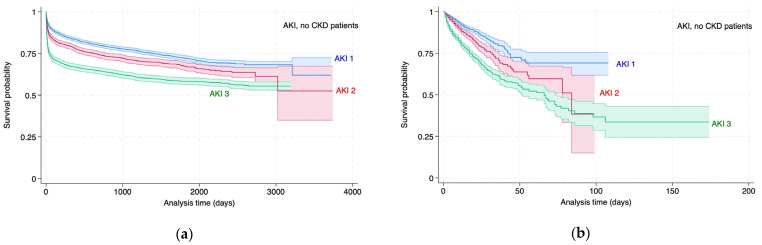
Kaplan–Meier estimates for AKI patients by stage, all-cause (**a**) and in-hospital (**b**) mortality.

**Figure 3:**
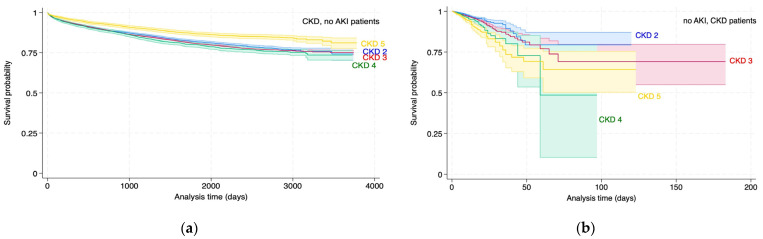
Kaplan–Meier estimates for CKD patients by stage, all-cause (**a**) and in-hospital (**b**) mortality.

Survival analysis of KD status by stage (AKI 1–3, CKD 1–5) for all-cause and in-hospital mortality was conducted to analyse the difference in outcomes between the clinically defined patient groups. The Kaplan–Meier estimates are represented in Figure 2 and Figure 3 and Table 3 and Table 4. The analysis confirms (1) the expected increase in mortality risk associated with Acute Kidney Injury (AKI) and Chronic Kidney Disease (CKD) as the disease progresses through its stages and (2) the external validation of the DDD rule engine’s output, as well as the general accuracy of the coding.

These results are interpreted as follows: Patients with AKI2 have a higher all-cause mortality risk of 28% (±12%), and patients with AKI3 have a higher all-cause mortality risk of 86% (±16%) relative to the AKI1 stage. Patients with AKI2 have higher in-hospital mortality risks of 53% (±26%) and 161% (±36%) relative to the AKI1 stage.

These results are interpreted as follows: Patients with CKD3 and CKD4 have a higher all-cause mortality risk relative to CKD2. Patients with CKD5 have a lower all-cause mortality risk relative to CKD2. Patients with CKD3, CKD4 and CKD5 have a higher in-hospital mortality risk relative to CKD2.

### 3.3. Survival Analysis of KD Staging Status

**Figure 4:**
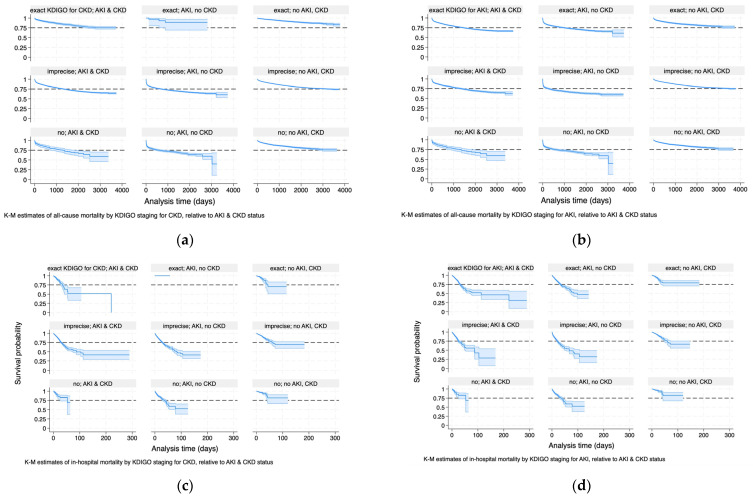
Kaplan–Meier estimates for all-cause and in-hospital mortality of KDIGO staging for AKI and CKD relative to kidney disease status. S = 0.75 is added as a reference line for ease of interpretability. (**a**) All-cause, CKD staging; (**b**) all-cause, AKI staging; (**c**) in-hospital, CKD staging; (**d**) in-hospital, AKI staging.

Survival analysis of KD staging status (exact AKI, exact CKD, imprecise and no staging) by KD status (AKI_noCKD, noAKI_CKD, AKI_CKD) for in-hospital and all-cause mortality was conducted to analyse whether there was a difference in outcomes between the clinically equivalent patient groups by staging status. The Kaplan–Meier estimates are represented in Figure 4 and Table 5 and Table 6.

These results are interpreted as follows:

*Imprecise* and no staging for CKD have higher all-cause mortality risks of 85% (±16%) and 84% (±18%) relative to exact CKD staging for any given disease status. Compared to acute-on-chronic KD (AKI_CKD), AKI (AKI_noCKD) has a higher all-cause mortality risk (27%, ±6%). The all-cause mortality risk for CKD without superimposed episodes of AKI (noAKI_CKD) is reduced by 39% (±26%).
*Imprecise* and no staging for AKI have higher all-cause mortality risks of 16% (±6%) and 10% (±8%) relative to exact AKI staging for any given disease status. Compared to acute-on-chronic KD (AKI_CKD), AKI (AKI_noCKD) has a higher all-cause mortality risk (32%, ±6%). The all-cause mortality risk for CKD without superimposed episodes of AKI (noAKI_CKD) is reduced by 43% (±2%).

These results are interpreted as follows:

*Imprecise* staging for CKD has a higher in-hospital mortality risk of 23% (±2%) relative to exact CKD staging for any given disease status. The difference in no staging is statistically non-significant. Compared to acute-on-chronic KD (AKI_CKD), AKI (AKI_noCKD) has a higher in-hospital mortality risk (30%, ±10%). The in-hospital mortality risk for CKD without superimposed episodes of AKI (noAKI_CKD) is reduced by 59% (±4%).
*Imprecise* and no staging for AKI have a higher in-hospital mortality risk of 48% (±2 × 0.06) relative to exact AKI staging for any given disease status. The difference in no AKI staging is statistically non-significant. Compared to acute-on-chronic KD (AKI_CKD), AKI (AKI_noCKD) has a higher all-cause mortality risk (30%, ±2 × 0.05). The all-cause mortality risk for CKD without superimposed episodes of AKI (noAKI_CKD) is reduced by 66%, ±2 × 0.02.

In the following subsections and in Supplementary File S3, the analyses accounting for baseline and confounder effects are reported.

### 3.4. Survival Analysis Taking into Account Baseline Factors

Survival analysis, i.e., the Cox regression model with the Breslow method for ties, was conducted to investigate the impact of underlying foundational factors (age, sex, comorbidities) and the type of treatment on different groups (KD status staging, KD status). All of the results are available in Supplementary File S3, Tables S1–S8.

The Cox regression model for all-cause mortality shows a hazard ratio (HR) of 1.14 (95% CI: 1.09 to 1.20) for AKI staging imprecise and HR of 1.24 for AKI staging no (95% CI 1.16 to 1.33) compared with the exact KDIGO staging. Patients with AKI have a higher HR of 1.96 (95% CI: 1.86 to 2.06), and patients with CKD without an acute component (noAKI_CKD) have a lower HR (0.72, 95% CI 0.69 to 0.76) compared with the KD status acute-on-chronic (AKI_CKD). Sex is non-significant, and age is minimally significant with HR 1.05 (95% CI 1.05–1.05). Patients receiving nephrotoxic medication and contrast-media imaging have increased in-hospital mortality (HR 1.7, 95% CI 1.53, 1.9; HR 1.26, CI 1.1, 1.43) compared with patients who did not receive this type of treatment. The conditions such as admission to ICU and the treatment groups oncology, neurosurgery, thoracic surgery, pneumology and trauma have a higher in-hospital mortality risk and, therefore, a considerable prognostic effect on mortality while incorporating a leading medical problem and the provided treatment. The results for the treatment groups are similar for all KD stages and KD staging groups. The results for the patients with AKI only are similar to those for all patients. The imprecise and no AKI staging statuses have a larger effect on in-hospital mortality in this patient’s population: imprecise, HR 1.24, 95% CI 1.17 to 1.31, and no staging, HR 1.46, 95% CI 1.33 to 1.60, compared with exact AKI staging.

The results of CKD staging for CKD patients show that the imprecise (HR 1.31, 95% CI 1.20 to 1.43) and no (HR 1.47, 95% CI 1.31 to 1.65) CKD staging have a significant effect on in-hospital mortality compared with exact CKD staging.

The most significant effect of AKI staging can be shown for the group of patients with AKI only: imprecise staging, HR 1.56, 95% CI 1.43 to 1.70, and no staging, HR 1.57, 95% CI 1.37 to 1.81.

### 3.5. Chi-Squared Tests for the Selection Bias Based on Sex and Age

The KDIGO staging findings for the different disease groups were analysed to detect statistically significant differences between sex (m, w) and age and their combination (selection bias study). The distribution of cases is shown in Table 7.

#### 3.5.1. CKD Staging by Sex

There is a statistically significant association between the variables men (m) and women (w), where men have a higher proportion of exact staging but a lower proportion of imprecise and no staging compared to f than expected: AKI_CKD: Pearson χ^2^ = 24.68, degrees of freedom (df) = 2, *p* < 0.001; noAKI_CKD: Pearson χ^2^ = 110.2, df = 2, *p* < 0.001.

#### 3.5.2. AKI Staging by Sex

There is a statistically significant association between the variables men (m) and women (w), where men have a higher proportion of exact staging but a lower proportion of imprecise and no staging compared to women than expected: AKI_noCKD: Pearson χ^2^ = 9.45, df = 2, *N* = 10,911, *p* = 0.009.

#### 3.5.3. CKD Staging by Age

There is a statistically significant association between the age at admission, where the exact staging group is the youngest and imprecise the oldest: AKI_CKD: Bartlett’s equal-variances test χ^2^ = 45.21, degrees of freedom (df) = 2, *p* < 0.001; noAKI_CKD: Bartlett’s equal-variances test χ^2^ = 110.2, df = 2, *N* = 38,740, *p* < 0.001.

#### 3.5.4. AKI Staging by Age

There is a statistically significant association between the age at admission, where the no staging group is the youngest and imprecise the oldest: AKI_noCKD: Bartlett’s equal-variances test χ^2^ = 26.5, df = 2, *p* < 0.001.

#### 3.5.5. CKD Staging by Sex and Age

There are statistically significant age differences for all pairwise comparisons for the groups AKI_CKD and noAKI_CKD, holding for both m and w patients: exact are the youngest, and imprecise are the oldest. For the stage-specific KDIGO staging (CKD2, CKD3, CKD4), men have a higher proportion of exact and a lower proportion of no compared with women, as expected (CKD2 Pearson χ^2^ = 23.8, df = 2, *p* < 0.001; CKD3 Pearson χ^2^ = 113.7, df = 2, *p* < 0.001; CKD4 Pearson χ^2^ = 59.43, df = 2, *p* < 0.001). For CKD5, men have lower exact and no than expected and women higher exact and no (Pearson χ^2^ = 17.8, df = 2, *p* < 0.001).

#### 3.5.6. AKI Staging by Sex and Age

There is a statistically non-significant association between the age at admission, where the no staging group is the youngest and the exact and the imprecise are the oldest: AKI_noCKD: Bartlett’s equal-variances test χ^2^ = 4.8, degrees of freedom (df) = 2, *p* = 0.089. For the stage-specific KDIGO staging (AKI1, AKI2, AKI3), men have a statistically significant lower proportion of no compared with women, as expected (AKI1 Pearson χ^2^ = 8.35, df = 2, *p* = 0.015. For the AKI2 and AKI2, the differences are non-significant. For all AKI staging groups, no is the youngest. The exact and imprecise are always older, and the age difference is statistically non-significant.

### 3.6. Distribution of the Hospitalisations’ Costs

A one-way ANOVA test was conducted to analyse the total cost of hospitalisation distribution. The overall ANOVA test revealed a significant difference in cost distribution among the groups exact, imprecise and diagnosis missing, F value 179.4, df = 2, *p*-value < 0.001, where the highest cost is assigned for imprecise and the lowest to exact staging.

## 4. Discussion

These results support the hypothesis that exact KD staging is associated with lower in-hospital and all-cause mortality and lower hospitalisation costs. Patients with AKI experience a more significant benefit from improved staging than those without AKI. Major decisions require timely action, as the implications of making or neglecting the right course of action can be substantial and influence the outcome. The mechanism of how precise staging leads to better outcomes still needs to be fully understood. The effect might be explained through diligence and professionalism, which led to fewer iatrogenic events, as the patients in the exact KDIGO staging group experienced less application of nephrotoxic medication and contrast agents. This might indicate that considering KD through treatment and diagnostics leads to a chain of events that protects kidney function. While a documented diagnosis is available in the EHR and accessible to each involved health care professional, the professionalism of the treatment team can contribute to more accurate staging, which is often a byproduct of higher-quality treatment.

Assessment of selection bias indicates that men experience exact staging more often than the women, suggesting a potential gender disparity. Interestingly, sex is not a significant covariate in survival predictions, even if women have a higher life expectancy, considering a sex-specific KD staging [2,38,39,40]. The higher proportion of imprecise KD staging among women leads to a deviation from the expected outcome according to life expectancy. We also found selection bias concerning age, where older patients more often receive an imprecise staging compared to younger ones. These results help us to understand which patient groups might be the most vulnerable and where action is needed.

Considering the potential impact of the current staging and treatment practice of KD, we identified studies on longitudinal follow-up and outcomes among a population with chronic kidney disease in large, managed care organisations. The distribution of characteristics and results show similar outcomes, so there might be a general problem with the appropriate recognition, staging and treatment of KD in a hospital setting [17,41,42]. The update of the KDIGO Clinical Practice Guideline for the Evaluation and Management of Chronic Kidney Disease (CKD), published in 2024, emphasises the importance of early recognition and staging of CKD, providing evidence for the associations of all complications of CKD are incrementally increased with worsened categories of eGFR and albuminuria [43]. The authors describe “the necessity of having both eGFR and ACR parameters” as a crucial precondition for an individual risk assessment. In 2013, Tomonaga et al. estimated a CKD prevalence of 700,000 or 11.4% of the subjects older than 15 years in the Swiss population [44]. The post-COVID life expectancy in Switzerland for men is 81.0 years and 85.1 for women. Hence, an estimated 2.2 Years of Life Lost (YLL) for men and women in the KD cohort can be projected on the 10% of the Swiss population with CKD, affecting ca 800,000 people (prevalence of 10%) [45,46,47].

The strength of our study is the rich, validated data set, with high consistency in coding policy, data quality and system settings. It is based on a unique, validated, data-driven diagnostic approach to identifying and coding diagnoses. Supported by the rule-based data-driven approach, our study helps to determine the true prevalence of KD in a hospital setting. To our knowledge, our study is the first acute-on-chronic model that reflects the simultaneous effects of AKI and CKD. According to the recent KDIGO 2024 Clinical Practice Guidelines, we still need more evidence to determine “whether an intervention to detect, risk-stratify, and treat CKD would improve the health outcomes for the targeted population” [48]. Our study also has limitations. As a single-centre observational study from a tertiary university hospital, this study might not reflect the broader context or societal perspective, so the generalisability needs to be questioned. The context of inpatient hospitalisation and treatment is a central element of the study; therefore, the outpatient setting and home care are not reflected in the data—the lack of clinical parameters and simplification of the clinical path are among the foremost to mention. Further, we plan to analyse time-to-event variables concerning diagnostics, RRT start and end, admission to ICU and chronification, while considering the absolute laboratory and medication values and the disease trajectory.

Future research might focus on economic modelling to gain a more granular and precise simulation of the staging effects and account for uncertainties. Sugrue identified 101 economic models of CKD, pointing out the difficulty of reflecting the heterogeneity of the patients’ trajectories and the need to “improve economic modelling accuracy in CKD” while using a “classical” approach such as a Markov model utilising CKD stages and a linear GFR decline [49]. Using microsimulation, it might be possible to attain more precision for specific medical conditions and treatment paths, including chronification and acute-on-chronic conditions, considering the multidimensionality of comorbidities to develop clinical decision support systems and predictive models based on this knowledge [26,42,50,51,52,53,54,55,56,57,58,59,60,61].

## 5. Conclusions

Our study provides valuable insight into potential quality and outcome improvements. Considering elderly patients, women and patients with acute-on-chronic KD as the most vulnerable, improving staging might play an essential role in better treatment, amenable mortality and lower costs. To enhance precise staging in clinical practice, focusing on generating high-quality evidence and guidelines, supporting the implementation through leadership and clinical education is needed. Clinician and patient involvement, along with advanced tools for pattern recognition and non-intrusive alert systems, can streamline integration and foster continuous improvement in outcomes.

## Figures and Tables

**Table 1:** Descriptive statistics of the studied kidney disease patient cohort, 2016–2023.

Variable	Total	AKI_noCKD	noAKI_CKD	AKI_CKD
Total cases *N* (%)	63,105	10,911 (17.2)	38,740 (61.5)	13,454 (21.3)
Age, mean (SD ^1^)	74.06 (13.39)	67.68 (15.42)	75.11 (12.75)	76.23 (11.79)
Sex (w/m) *N* (%)	26,326/36,779 (42/58)	4223/6688	16,530/22210	5573/7881
Elixhauser van Walraven weighted mean (SD)	5.00 (2.18)	4.19 (2.11)	4.86 (2.03)	6.03 (2.27)
Tox. medication ^2^ yes/no, *N* (%)	6235/56,870 (10/90)	1682/9229 (15/85)	3169/35,571 (8/92)	1384/12,070 (10/90)
Contrast imaging yes/no, *N* (%)	2284/60,821 (4/96)	1032/9879 (9/91)	638/38,102 (2/98)	614/12,840 (5/95)
ICU admission yes/no, *N* (%)	7782/55,323 (12.3/87.7)	3193/7718 (29/71)	2440/36,300 (6/94)	2149/11,305 (16/84)
Total costs (SD)	26,112.03 (41,643)	45,193.40 (67204)	18,973.28 (23,801)	31,153.46 (48652)
Total costs/day (SD)	3615.88 (3862)	4043.03 (4770)	3706.63 (3800)	3008.70 (3067)
AKI Staging exact/imprec/no *N*	18,279/37,488/7338	6147/2960/1804	2973/30,581/5186	9159/3947/348
AKI Staging exact/imprec/no (%)	29/59/12	56/27/17	8/79/13	68/29/3
CKD Staging exact/imprec/no *N*	4564/51,396/7145	33/9074/1804	3290/30,453/4997	1241/11,869/344
CKD Staging exact/imprec/no (%)	7/81/11	0/83/17	8/79/13	9/88/3
All-cause mortality	13,742	3223	6902	3617
In-hospital mortality	3608	1426	953	1229

^1^ SD Standard Deviation, ^2^ toxic medication.

**Table 2:** Distribution of KD groups (AKI_CKD, AKI_noCKD, noAKI_CKD) in the most relevant treatment groups.

Treatment Group	AKI_CKD *n* (%)	AKI_noCKD *n* (%)	noAKI_CKD *n* (%)	Total *n* (%)
General Medicine	6121 (45)	3997 (37)	13,876 (36)	23,994 (38)
Cardiology	1205 (9)	854 (7.8)	5975 (15)	8034 (13)
Musculoskeletal	790 (6)	625 (5.7)	2403 (6)	3818 (6)
Pneumology	735 (5.5)	607 (5.6)	1079 (2.8)	2421 (3.8)
Haematology	654 (4.8)	607 (5.6)	1266 (3)	2527 (4)
Gastroenterology	580 (4.3)	467 (4)	1152 (3)	2199 (3.5)
Heart surgery	449 (3.3)	622 (6)	893 (2)	1964 (3)
Urology	417 (3)	545 (5)	1370 (4)	2332 (3.7)
Endocrinology	362 (2.7)	200 (2)	647 (1.7)	1209 (2)
Neurology	355 (2.6)	336 (3)	2075 (5.4)	2766 (4.4)
Nephrology	336 (2.5)	318 (3)	1273 (3)	1927 (3)
Vascular	312 (2.3)	328 (3)	1215 (3)	1855 (3)
Visceral Surgery	256 (2)	472 (4.3)	397 (1)	1125 (2)
Total	63,105 (100)	10,911 (100)	38,740 (100)	13,454 (100)

**Table 3:** Kaplan–Meier estimates for AKI patients by stage all-cause mortality (a) and in-hospital mortality (b), analysis referring to Figure 2.

Variable	Hazard Ratio	Standard Error	z	P > |z|	95% Confidence Interval
AKI stage, all-cause mortality (a)					
AKI1 (reference)					
AKI2	1.28	0.06	4.92	<0.001	1.16 to 1.41
AKI3	1.86	0.08	14.43	<0.001	1.71 to 2.02
AKI stage, in-hospital mortality (b)					
AKI1 (reference)					
AKI2	1.54	0.13	5.22	<0.001	1.31 to 1.81
AKI3	2.61	0.18	14.12	<0.001	2.29 to 2.99

**Table 4:** Kaplan–Meier estimates for CKD patients by stage all-cause mortality (a) and in-hospital mortality (b), analysis referring to Figure 3.

Variable	Hazard Ratio	Standard Error	z	P > |z|	95% Confidence Interval
CKD stage, all-cause mortality (a)					
CKD2 (reference)					
CKD3	1.05	0.03	1.7	0.089	0.99 to 1.12
CKD4	1.11	0.05	2.2	0.028	1.01 to 1.21
CKD5	0.68	0.04	−7.17	<0.001	0.61 to 0.75
CKD stage, in-hospital mortality (b)					
CKD2 (reference)					
CKD3	1.38	0.12	0.65	<0.001	1.16 to 1.65
CKD4	1.76	0.23	4.38	<0.001	1.37 to 2.27
CKD5	2.47	0.29	7.68	<0.001	1.96 to 3.11

**Table 5:** Kaplan–Meier estimates for all-cause mortality of CKD and AKI staging status, analysis referring to Figure 4a,b.

Variable	Hazard Ratio	Standard Error	z	P > |z|	95% Confidence Interval
CKD staging status, all-cause mortality					
Exact CKD (reference)					
Imprecise CKD	1.85	0.08	14.31	<0.001	1.7 to 2.01
No CKD	1.84	0.09	11.97	<0.001	1.66 to 2.03
KD status					
AKI_CKD (reference)					
AKI_noCKD	1.27	0.031	9.63	<0.001	1.21 to 1.33
noAKI_CKD	0.61	0.013	−23.47	<0.001	0.59 to 0.64
AKI staging status, all-cause mortality					
Exact AKI (reference)					
Imprecise AKI	1.16	0.03	6.57	<0.001	1.11 to 1.21
No AKI	1.1	0.04	3.01	0.003	1.04 no 1.18
KD status					
AKI_CKD (reference)					
AKI_noCKD	1.32	0.03	11.14	<0.001	1.25 to 1.38
noAKI_CKD	0.57	0.01	−23.35	<0.001	0.54 to 0.59

**Table 6:** Kaplan–Meier estimates for in-hospital mortality of CKD and AKI staging status, analysis referring to Figure 4c,d.

Variable	Haz. Ratio	Standard Error	z	P > |z|	95% Confidence Interval
CKD staging status, in-hospital mortality					
Exact CKD (reference)					
Imprecise CKD	1.23	0.1	2.48	0.013	1.20 to 1.41
No CKD	0.97	0.1	−0.28	0.78	0.80 to 1.18
KD status					
AKI_CKD (reference)					
AKI_nonoCKD	1.3	0.05	6.46	<0.001	1.21 to 1.33
noAKI_CKD	0.41	0.02	−19.92	<0.001	0.38 to 0.45
AKI staging status, in-hospital mortality					
Exact AKI (reference)					
Imprecise AKI	1.48	0.06	9.78	<0.001	1.37 to 1.60
No AKI	0.97	0.06	−0.56	0.57	0.86 to 1.09
KD status					
AKI_CKD (reference)					
AKI_noCKD	1.3	0.05	6.5	<0.001	1.20 to 1.41
noAKI_CKD	0.34	0.02	−22.33	<0.001	0.30 to 0.37

**Table 7:** KD stage groups and CKD staging, distribution of cases.

KD Group	Exact	Imprecise	no	Total
CKD staging ^1^				
AKI_CKD	1241	11,869	344	13,454
AKI_noCKD	33	9074	1804	10,911
noAKI_CKD	3290	30,453	4997	38,740
Total	4564	51,396	7145	63,105
AKI staging ^2^				
AKI_CKD	9159	3947	348	13,454
AKI_noCKD	6147	2960	1804	10,911
noAKI_CKD	2973	30,581	5186	38,740
Total	18,279	37,488	7338	63,105

^1^ Pearson χ^2^ = 2300, df = 4, *p* < 0.001; ^2^ Pearson χ^2^ = 24,000, df = 4, *p* < 0.001.

## Data Availability

The data presented in this study are available only on request from the corresponding author and restricted due to legal reasons.

## Supplementary Materials

The following supporting information can be downloaded at: https://www.mdpi.com/article/10.3390/diagnostics14222476/s1, File S1: ICD-10; File S2: Terms for the text search; File S3: Cox regression models.
