# Supplementary material for: Impact of Precision in Staging Acute Kidney Injury and Chronic Kidney Disease on Treatment Outcomes: An Observational Study
Source: Diagnostics (Basel). 2024 Nov 6;14(22):2476. doi: 10.3390/diagnostics14222476 (PMC11592415; doi:10.3390/diagnostics14222476)
Supplement: Supplementary file 1 [file diagnostics-14-02476-s001.zip › Supplement File S1.pdf]

**ICD-10**	**KD definition**
N17.0; N17.1; N17.2; N17.8; N17.9; "RIFLE3 2016"	AKI3_RIFLE
N17.x1 "AKI1"	AKI1
N17.x2 "AKI2"	AKI2
N17.x3 "AKI3"	AKI3
N17.x9 "AKIna"	AKIna
N18.1 "CKD1"	CKD1
N18.2 "CKD2"	CKD2
N18.3 "CKD3"	CKD3
N18.4 "CKD4"	CKD4
N18.5 "CKD5"	CKD5
N18.80 "CKD rest kidney"	CKD_rest kidney
N18.89; N18.9 "CKDna"	CKDna
N19	KDna
