# Supplementary material for: Impact of Precision in Staging Acute Kidney Injury and Chronic Kidney Disease on Treatment Outcomes: An Observational Study
Source: Diagnostics (Basel). 2024 Nov 6;14(22):2476. doi: 10.3390/diagnostics14222476 (PMC11592415; doi:10.3390/diagnostics14222476)
Supplement: Supplementary file 1 [file diagnostics-14-02476-s001.zip › Supplement File S2.pdf]

The «KD Staging imprecise» search term is defined as “acute-on-chronic Niereninsuffizienz”, “acute Niereninsuffizienz”, “akute Niereninsuffizienz”, “chronische Niereninsuffizienz”, “Niereninsuffzienz”, “Nierenversagen”, “renal insufficiency”, “RIFLE”, “AKI”, “CKD”, “eGFR”, “KDIGO”, “Kreatinin”, “Creatinin”. The “KD Staging missing” was applied if none of the “KD Staging imprecise” terms were found. A fuzzy search algorithm was applied for terms with a tilde character (“~”) to ensure that not only one spelling of a term was found. The extraction used TheFuzz, RapidFuzz libraries, packages numpy, panda, release 23.3.1, Python, Visual Studio September 2023, version 1.83.
