# Supplementary material for: Impact of Precision in Staging Acute Kidney Injury and Chronic Kidney Disease on Treatment Outcomes: An Observational Study
Source: Diagnostics (Basel). 2024 Nov 6;14(22):2476. doi: 10.3390/diagnostics14222476 (PMC11592415; doi:10.3390/diagnostics14222476)
Supplement: Supplementary file 1 [file diagnostics-14-02476-s001.zip › Supplement File S3.pdf]

Table S1. Cox regression model for all-cause mortality, AKI staging, including the treatment groups

Variable	Hazard ratio	Standard error	z	P>|z|	95% confidence interval
exact_AKI (reference)					
imprecise_AKI	1.14	0.03	5.84	<0.001	1.09 to 1.20
no	1.24	0.04	6.44	<0.001	1.16 to 1.33
AKI_CKD (reference)					
AKI_noCKD	1.96	0.05	25.32	<0.001	1.86 to 2.06
noAKI_CKD	0.72	0.02	-12.84	<0.001	0.69 to 0.76
sex f	1.00	0.02	0.05	0.957	0.97 to 1.04
age_entry	1.05	0.00	54.77	<0.001	1.05 to 1.05
tox_med	1.29	0.04	7.73	<0.001	1.21 to 1.38
ICU	2.19	0.06	29.34	<0.001	2.08 to 2.31
Elixhauser scores unweighted	0.92	0.01	-14.86	<0.001	0.91 to 0.93
Elixhauser van Walraven weighted	1.05	0.00	38.75	<0.001	1.05 to 1.06
contrast_imaging	1.01	0.05	0.14	0.890	0.92 to 1.10
Treatment*					
Cardiology	0.95	0.07	-0.67	0.505	0.83 to 1.10
General Medicine	1.12	0.08	1.7	0.090	0.98 to 1.29
Heart surgery	0.55	0.05	-6.72	<0.001	0.46 to 0.66
Musculoskeletal	0.87	0.07	-1.78	0.075	0.75 to 1.01
Nephrology	1.19	0.10	2.09	0.036	1.01 to 1.41
Neurology	1.34	0.10	3.85	<0.001	1.16 to 1.56
Neurosurgery	1.77	0.16	6.17	<0.001	1.48 to 2.12
Oncology	2.02	0.21	6.61	<0.001	1.64 to 2.48
Pneumology	1.54	0.12	5.6	<0.001	1.32 to 1.79
Postacute	0.64	0.08	-3.65	<0.001	0.50 to 0.81
Rheumatology	0.67	0.13	-2.13	0.034	0.47 to 0.97
Thoracic surgery	1.79	0.26	3.96	<0.001	1.34 to 2.38
Transplantation	0.31	0.06	-6.17	<0.001	0.21 to 0.45
Trauma	1.45	0.16	3.31	0.001	1.16 to 1.81
Urology	0.84	0.07	-2.05	0.040	0.70 to 0.99
Visceralsurgery	1.21	0.11	2.12	0.034	1.01 to 1.43

*Treatment group Angiologie wa taken as a reference; Likelihood ratio $\chi^2(38) = 8669.74$, $p < 0.001$, N = 62736, N events = 13644

Table S2. Cox regression model for all-cause mortality, AKI staging, AKI patients only

Variable	Hazard ratio	Standard error	z	P>|z|	95% confidence interval
exact AKI (reference)					
imprecise_AKI	1.24	0.04	7.63	<0.001	1.17 to 1.31

no	1.46	0.07	8.07	<0.001	1.33 to 1.60
AKI_CKD				<0.001	
AKI_noCKD	1.70	0.05	18.55	<0.001	1.61 to1.80
AKI Stage					
AKI2	1.33	0.05	8.18	<0.001	1.24 to1.42
AKI3	1.80	0.06	17	<0.001	1.68 to 1.92
sex f	1.01	0.03	0.47	0.637	0.96 to 1.06
age_entry	1.04	0.00	35.78	<0.001	1.04 to 1.05
tox. Medication	1.22	0.05	5.03	<0.001	1.13 to1.33
ICU	1.86	0.06	18.17	<0.001	1.74 to 1.99
contrast_imaging	0.93	0.05	-1.37	0.171	0.85 to 1.03

Likelihood ratio $\chi^2(42) = 3755.45$, $p < 0.0000$, N = 24011, N events = 6757

Table S3. Cox regression model for all-cause mortality, CKD staging, all patients

Variable	Hazard ratio	Standard error	z	P>|z|	95% confidence interval
exact_CKD reference					
imprecise	1.35	0.06	6.87	<0.001	1.24 to 1.47
no	1.53	0.08	8.24	<0.001	1.38 to 1.69
AKI_CKD reference				<0.001	
AKI_noCKD	1.93	0.05	24.69	<0.001	1.83 to 2.03
noAKI_CKD	0.78	0.02	-11.52	<0.001	0.75 to 0.81
sex f	1.00	0.02	-0.1	0.917	0.96 to 1.03
age_entry	1.05	0.00	54	<0.001	1.05 to 1.05
tox medication	1.29	0.04	7.78	<0.001	1.21 to 1.38
ICU	2.18	0.06	29.23	<0.001	2.07
contrast imaging	1.01	0.05	0.25	0.799	0.93

Likelihood ratio $\chi^2(42) = 8690.35$, $p < 0.001$, N = 62736, N events = 13644

Table S4. Cox regression model for all-cause mortality, CKD staging, CKD patients only

Variable	Hazard ratio	Standard error	z	P>|z|	95% confidence interval
exact CKD (reference)					
imprecise_CKD	1.31	0.06	6.14	<0.001	1.20 to 1.43
no	1.47	0.09	6.63	<0.001	1.31 to 1.65
AKI_CKD (reference)				<0.001	
noAKI_CKD	0.77	0.02	-11.68	<0.001	0.74 to 0.81
ckdstage					
CKD2	0.92	0.11	-0.71	0.481	0.72 to 1.17
CKD3	0.85	0.10	-1.31	0.192	0.67 to 1.08
CKD4	0.98	0.12	-0.12	0.901	0.77 to 1.26
CKD5	1.17	0.16	1.21	0.226	0.91 to 1.52
CKDna	1.49	0.19	3.12	0.002	1.16 to 1.92
sex f	0.99	0.02	-0.33	0.744	0.95 to 1.03
age_entry	1.06	0.00	49.78	<0.001	1.06 to 1.06
tox_med	1.26	0.05	5.26	<0.001	1.16 to 1.37

icu	2.13	0.07	22.6	<0.001	2.00 to 2.28

Likelihood ratio $\chi^2(42) = 6391.89$, $p < 0.0000$, N = 52164, N events = 10489

Table S5. Cox regression model for in-hospital mortality, AKI staging, all patients

Variables	Hazard ratio	Standard error	z	P>|z|	95% confidence interval
exact AKI (reference)					
imprecise_AKI	1.44	0.06	9.03	<0.001	1.33 to 1.56
no	1.09	0.07	1.29	0.195	0.96 to 1.23
AKI_CKD (reference)					
AKI_noCKD	1.40	0.06	7.53	<0.001	1.28 to 1.53
noAKI_CKD	0.44	0.02	-16.04	<0.001	0.40 to 0.49
sex f	0.94	0.03	-1.79	0.073	0.87 to 1.01
age_entry	1.03	0.00	20.5	<0.001	1.03 to 1.04
tox medication	0.95	0.05	-1.1	0.272	0.86 to 1.04
ICU	3.38	0.15	26.56	<0.001	3.09 to 3.69
contrast imaging	0.80	0.05	-3.69	<0.001	0.71 to 0.90

Likelihood ratio $\chi^2(42) = 3352.99$, $p < 0.0000$, N = 62583, N events = 3453

Table S6. Cox regression model for in-hospital mortality, AKI staging, AKI patients only

Variables	Hazard ratio	Standard error	z	P>|z|	95% confidence interval
exact AKI (reference)					
imprecise	1.56	0.07	9.9	<0.001	1.43 to 1.70
no	1.57	0.11	6.39	<0.001	1.37 to 1.81
AKI_CKD (reference)				<0.001	
AKI_noCKD	1.25	0.06	4.74	<0.001	1.14 to 1.37
sex w	0.97	0.04	-0.61	0.540	0.90 to 1.06
akistage					
AKI2	1.59	0.09	7.86	<0.001	1.42 to 1.79
AKI3	2.45	0.13	16.29	<0.001	2.20 to 2.72
age_entry	1.03	0.00	18.11	<0.001	1.03 to 1.04
tox_med	0.89	0.05	-2.19	0.028	0.79 to 0.99
contrast_Imaging	0.77	0.05	-4.06	<0.001	0.67 to 0.87

Likelihood ratio $\chi^2(42) = 1955.73$, $p < 0.0000$, N = 23,939, N events = 2,545

Table S7. Cox regression model for in-hospital mortality, CKD staging, all patients

Variables	Hazard ratio	Standard error	z	P>|z|	95% confidence interval
Exact_CKD (reference)					
imprecise_CKD	0.98	0.08	-0.28	0.783	0.83 to 1.15
no	0.88	0.09	-1.3	0.195	0.72 to 1.07
AKI_CKD (reference)					
AKI_noCKD	1.42	0.06	7.82	<0.001	1.30 to 1.56

noAKI_CKD	0.54	0.02	-13.35	<0.001	0.49 to 0.59
sex w	0.94	0.03	-1.6	0.110	0.88 to 1.01
age_entry	1.03	0.00	20.59	<0.001	1.03 to 1.04
tox_med	0.96	0.05	-0.87	0.386	0.87 to 1.06
icu	3.40	0.16	26.74	<0.001	3.10 to 3.71
contrast_imaging	0.80	0.05	-3.57	<0.001	0.71 to 0.91

Likelihood ratio $\chi^2(42) = 3272.75$, $p < 0.0000$, N = 62,583, N events = 3,453

Table S8. Cox regression model for in-hospital mortality, CKD staging, CKD patients only

Variable	Hazard ratio	Standard error	z	P>|z|	95% confidence interval
exact CKD (reference)					
imprecise_CKD	1.08	0.09	0.88	0.376	0.91 to 1.28
no	0.82	0.11	-1.41	0.159	0.63 to 1.08
AKI_CKD (reference)					
noAKI_CKD	0.56	0.03	-11.88	<0.001	0.51 to 0.62
sex f	0.89	0.04	-2.48	0.013	0.81 to 0.98
CKD stage					
CKD2	0.93	0.29	-0.22	0.826	0.51 to 1.70
CKD3	1.07	0.32	0.22	0.824	0.59 to 1.94
CKD4	1.53	0.47	1.39	0.166	0.84 to 2.81
CKD5	2.52	0.80	2.93	0.003	1.36 to 4.69
age_entry	1.04	0.00	15.86	<0.001	1.03 to 1.04
tox_med	0.99	0.07	-0.13	0.895	0.86 to 1.14
icu	3.39	0.20	20.29	<0.001	3.01 to 3.81
contrast_imaging	0.87	0.08	-1.52	0.128	0.73 to 1.04

Likelihood ratio $\chi^2(42) = 2060.11$, $p < 0.0000$, N = 52039, N events = 2097
